# Late sodium current blocker GS967 inhibits persistent currents induced by familial hemiplegic migraine type 3 mutations of the *SCN1A* gene

**DOI:** 10.1186/s10194-019-1056-2

**Published:** 2019-11-15

**Authors:** R. Barbieri, S. Bertelli, M. Pusch, P. Gavazzo

**Affiliations:** 1 Biophysics Institute, National Research Council, Via De Marini 6, Genoa, Italy; 2 International School of Advanced Studies (SISSA), Via Bonomea, 265 Trieste, Italy

**Keywords:** FHM3, Voltage gated Na^+^ channel, Persistent current, Na^+^ current inactivation, Migraine treatment

## Abstract

**Background:**

Familial hemiplegic migraine (FHM) is a group of genetic migraine, associated with hemiparesis and aura. Three causative different genes have been identified, all of which are involved in membrane ion transport. Among these, *SCN1A* encodes the voltage-gated Na^+^ channel Nav1.1, and FHM caused by mutations of *SCN1A* is named FHM3. For 7 of the 12 known FHM3-causing *SCNA1* mutations functional consequences have been investigated, and even if gain of function effect seems to be a predominant phenotype, for several mutations conflicting results have been obtained and the available data do not reveal a univocal FHM3 pathomechanism.

**Methods:**

To obtain a more complete picture, here, we characterized by patch clamp approach the remaining 5 mutations (Q1489H, I1498M, F1499 L, M1500 V, F1661 L) in heterologous expression systems.

**Results:**

With the exception of I1498M, all mutants exhibited the same current density as WT and exhibited a shift of the steady state inactivation to more positive voltages, an accelerated recovery from inactivation, and an increase of the persistent current, revealing that most FHM3 mutations induce a gain of function. We also determined the effect of GS967, a late Na^+^ current blocker, on the above mentioned mutants as well as on previously characterized ones (L1649Q, L1670 W, F1774S). GS967 inhibited persistent currents of all SCNA1 FMH3-related mutants and dramatically slowed the recovery from fast inactivation of WT and mutants, consistent with the hypothesis that GS967 specifically binds to and thereby stabilizes the fast inactivated state.

Simulation of neuronal firing showed that enhanced persistent currents cause an increase of ionic fluxes during action potential repolarization and consequent accumulation of K^+^ and/or exhaustion of neuronal energy resources. In silico application of GS967 largely reduced net ionic currents in neurons without impairing excitability.

**Conclusion:**

In conclusion, late Na^+^ current blockers appear a promising specific pharmacological treatment of FHM3.

## Background

Familial hemiplegic migraine (FHM) is a severe form of hereditary autosomal dominant migraine with aura caused by mutations in three different genes encoding neuronal or glial ion transporting membrane proteins. FHM1 is caused by mutations in the gene encoding the presynaptic neuronal Ca^2+^ channel Cav2.1, FHM2 is associated with mutations of a glial specific Na-K-ATPase, whereas FHM3 is caused by mutations in *SCN1A*, the gene encoding the neuronal CNS specific Na^+^ channel Nav1.1 [1, 2]. While FHM is more severe than “common” migraine with aura, it is believed that the molecular mechanisms underlying FHM could provide insight into the pathophysiology of more general forms of migraine. The fact that all forms of FHM are associated with ion transporting neuronal/glial specific proteins hints to a “neuronal origin” of FHM and to a disturbance of ionic homeostasis at the basis of the disease. In agreement with this general assumption, the phenomenon of cortical spreading depression (CSD), which is characterized by a wave of K^+^ accumulation and generalized neuronal depolarization, has been proposed to be an essential event in migraine with aura [1]. *SCN1A* is a well-known epilepsy gene with hundreds of mutations being causative for various forms of epilepsy [3–5]. The predominant expression of *SCN1A* in inhibitory interneurons is in agreement with the finding that most epilepsy mutations reduce Nav1.1 functionality [3, 4]. Comparably, a much smaller number of *SCN1A* mutations has been found to be linked to FHM3 (12 mutations so far), and the genotype-phenotype correlation for migraine mutations is not fully clear. Several functionally studied mutations exhibit defects of the fast inactivation process such as a rightward shift of the steady-state inactivation, accelerated recovery from inactivation, and enhanced persistent currents [2, 6–14]. This finding is consistent with the hypothesis that FHM3 is associated with interneuron hyperexcitability. On the other hand, conflicting results have been reported for the properties of the mutations L1649Q, Q1489K, L263 V, and T1174S, when expressed in heterologous systems [7, 8, 11]. Thus, their characteristics are not fully clear. Finally, some FHM3 mutations result in reduced expression levels in heterologous expression systems [8, 10, 13, 14]. For these variants, the exact functional impact in patients cannot be fully predicted, even though an overall gain of function is most likely [10]. Based on these considerations, it appears extremely important to extend the functional investigation to all known FHM3-linked mutations to arrive at a complete picture.

Gain of function mutations with impaired fast inactivation in the muscle Na^+^ channel gene *SCN4A* lead to muscle hyperexcitability and myotonia [15] and in the cardiac *SCN5A* gene to Long QT (LQTS) and Brugada syndrome (BS) [16]. For LQTS and BS, “late Na^+^ current blockers”, like ranolazine, have been proposed as specific correctors of mutation-induced disruption of inactivation [17]. Hence, a similar strategy might be applicable in FHM3 patients.

Also GS697, a late Na^+^ current blocker, has been shown to exert a potent antiarrhythmic effect on rabbit myocytes and on canine or porcine models, targeting Nav1.5 heart channels [18–20]. In addition, GS967 administered as a possible therapeutic compound in various epileptic mouse models with mutations in Nav genes caused a strong reduction of spontaneous seizures and improved survival of affected mice [21–23]. The collective evidence suggests that the beneficial effect of GS967 is mediated at the molecular level by the block of unusually large persistent Na^+^ currents in cardiac myocytes and neurons in the different mouse models.

In the present study, in order to further test the generality of the assumption that FHM3 is associated with a gain of function, we examined the effects of five so far uncharacterized *SCNA1* mutations in heterologous expression systems, confirming for four of them a defective inactivation process. One mutation (I1498M) exhibited dramatically reduced functional expression. We then tested the effect of the late Na^+^ current blocker GS967 [24] on these four inactivation impaired mutations and on several previously characterized mutations. GS967 drastically reduced persistent currents and markedly delayed recovery from inactivation for WT and mutants. Simulation of neuronal firing properties suggests that enhanced persistent currents do not strongly affect excitability per se, but markedly increase net ionic transmembrane fluxes during action potential repolarization, predicting an increase of the extracellular K^+^ concentration and an important energetic burden on firing neurons. Is noteworthy that simulating a reduction of persistent current similar to that experimentally obtained by the application of GS697 ameliorated these effects without compromising excitability.

## Methods

### Chemicals

All salts were of highest available grade and were obtained from Sigma-Aldrich (Milan, Italy). GS967 was obtained from MedChemtronica AB The European branch of MCE (Sweden). Stock solution aliquots at 2 mM in DMSO were kept at − 20 °C and diluted in the experimental solution at the day of the experiment.

### Molecular biology

For heterologous expression of WT and mutant *SCN1A* we employed our previously characterized optimized *SCN1A* expression plasmid which encodes the shorter *SCN1A* splice variant and in which codons are changed to render the cDNA more similar to the cardiac SCN5A isoform, and which is much easier to handle than traditional *SCNA1* expression plasmids [14]. Expression of this optimized plasmid results in ionic currents that are indistinguishable from those seen after expression of the original *SCNA1* construct [14]. All new mutations (Q1489H, I1498M, F1499 L, M1500 V, F1661 L) were introduced by restriction free mutagenesis as described earlier [14] and the open reading frame was confirmed by Sanger sequencing. For expression in *Xenopus* oocytes the original pCDNA3-based constructs were transferred into the pFrog vector [25] using HindIII and XbaI restriction enzymes (Thermo Fisher, Milan, Italy). In *Xenopus* oocytes we co-expressed *SCN1A* with the human β1 subunit which is known to reduce slow components of inactivation [26]. The β1 subunit was cloned in the pGEM vector (kindly provided by Stephen Cannon). In HEK cells, experiments were carried out in the absence of the β1 subunit because functional effects of β1 subunit are generally rather small [10]. In addition, co-transfection of α and β subunits invariably leads to an uncontrolled variable stoichiometry, which might increase the variability of the data. In addition, all mutations studied here are far away from the α-β interaction surface as inferred from the eel structure [27].

### Cell culture and transfection

HEK-293 cells were grown and transfected as described earlier [14]. Briefly, we used DMEM supplemented with 10% FBS, penicillin, and streptomycin and maintained cells at 37 °C in 5% CO_2_, and 95% humidity. For transfection we used the Effectene Kit (Qiagen), co-transfecting 500 ng *SCN1A* plasmid and 50 ng of a vector expressing GFP. Currents were recorded 24-48 h after transfection.

### Oocyte injection

For expression in *Xenopus* oocytes, cRNA was transcribed by the mMessage mMachine T7 kit (Life Technologies, Milan, Italy) after linearization with Mlu I (SCNA1) or NheI (β1). cRNA (5 ng of *SCN1A* + 5 ng of β1) was injected in defolliculated oocytes, which were incubated at 18 °C in the maintaining solution containing (in mM): 90 NaCl, 2 KCl, 1 MgCl_2_, 1 CaCl_2_, and 10 Hepes (pH 7.5).

### Current recordings

Currents in HEK cells were recorded using the whole-cell configuration of the patch clamp technique [28] using an Axopatch 200B amplifier at room temperature (22–25 °C) as described earlier [14]. Bath solution contained (in mM): 145 NaCl, 5 KCl, 1.8 CaCl_2_, 1 MgCl_2_, 10 Hepes, pH 7.4. Internal solution contained 40 CsCl, 10 NaCl, 80 CsF, 11 EGTA, 10 Hepes, 1 CaCl_2_, pH 7.3. Cell capacitance was between 10 and 25 pF. We controlled that series resistance, R_S_, was not larger than 4 MΩ and often it was around 2 MΩ, for which a maximum accepted current level would be 2.5 nA to keep the voltage error smaller than 5 mV. The current levels shown in Fig. 2 (which are indeed representative) do not exceed 1.5 nA, being thus actually on the safe side.

Currents in *Xenopus* oocytes were measured using the two-electrode voltage clamp technique (TEVC) using an npi-electronics amplifier (Tamm, Germany) as described previously [29]. To reduce the current magnitude and to minimize voltage-clamp errors, in the TEVC experiments the extracellular solution contained a low concentration of NaCl. It had the following composition (in mM): 15 NaCl, 90 N-methyl-D-Glucamine-Cl, 10 Hepes, 2 CaCl_2_, 2 MgCl_2_, pH 7.3.

All measurements were performed using the custom acquisition program GePulse (available at http://users.ge.ibf.cnr.it/pusch/programs-mik.htm). Between the application of pulse protocols, the membrane was kept at a holding potential of − 90 mV (See Supplementary Methods).

### Statistical analysis

To evaluate statistical significance Students unpaired t-test was used. Significance levels were: not significant: *p* > =0.05; *: *p* < 0.05; **: *p* < 0.01; ***: *p* < 0.001.

*Modelling of Na* ^*+*^*channel gating with GS967 binding and simulation of neuronal firing.*

Gating of the sodium channel was modelled as described earlier [10, 11, 30] as

$$ {I}_{Na}={G}_{Na}\left(V-{E}_{Na}\right){m}^3 hs $$

where *I*_*Na*_ is the Na^+^ current, *G*_*Na*_ is the maximal conductance, *V* the membrane potential, *E*_*Na*_ the Na^+^ equilibrium potential (assumed to be 50 mV throughout), *m* the activation variable, *h* the fast inactivation variable and *s* the slow inactivation variable. Activation (*m*) and slow inactivation (*s*) variables were parameterized as in [10, 11]. The fast inactivation variable, *h*, is usually modelled as a two state process

$$ A{\displaystyle \begin{array}{c}{\alpha}_h\\ {}\leftrightarrows \\ {}{\beta}_h\end{array}}I $$

in which *A* (“activated”) denotes the non-inactivated state, *I* the inactivated state, *β*_*h*_ the inactivation rate constant and *α*_*h*_ the rate constant of recovery from inactivation. The standard inactivation rate constants *α*_*h*_ and *β*_*h*_ were calculated as in [10, 11].

In order to implement the assumption that GS967 binds exclusively to the inactivated state, we modelled inactivation as a three state process:

$$ A{\displaystyle \begin{array}{c}{\alpha}_h\\ {}\leftrightarrows \\ {}{\beta}_h\end{array}}I{\displaystyle \begin{array}{c}\mu \\ {}\leftrightarrows \\ {}\lambda \left[ GS967\right]\end{array}}{I}_{GS} $$

Here, *I*_*GS*_ represents the inactivated channel with a molecule of GS967 bound. Binding of GS967, and unbinding rate constants were assumed to have the values: λ = 10 μM^− 1^ s^− 1^, μ = 1 s^− 1^.

Further, in order to impose a certain “percentage of persistent current” in the absence of GS967, *P*_*persist*_ induced by a mutation, the inactivation rate constants (*β*_*h*_ and *α*_*h*_) were modified to ${\beta}_h^{\prime }$ and ${\alpha}_h^{\prime }$, respectively, based on the following equations.

In the standard model, steady state inactivation, *h*_∞_, is given by

$$ {h}_{\infty }=\frac{\alpha_h}{\alpha_h+{\beta}_h} $$

and approaches 0 at positive voltages, i.e. the standard model exhibits negligible persistent currents.

To introduce *P*_*persist*_ we defined the modified steady state inactivation, ${h}_{\infty}^{\prime }$, as

$$ {h}_{\infty}^{\prime }={P}_{persist}+\left(1-{P}_{persist}\right){h}_{\infty } $$

and obtained the modified inactivation rate constant, ${\beta}_h^{\prime }$, by

$$ {\beta}_h^{\prime }=\left(1-{h}_{\infty}^{\prime}\right)\left({\alpha}_h+{\beta}_h\right)=\left(1-{P}_{persist}\right){\beta}_h $$

and the modified recovery from inactivation rate constant, ${\alpha}_h^{\prime }$, by

$$ {\alpha}_h^{\prime }={h}_{\infty}^{\prime}\left({\alpha}_h+{\beta}_h\right)={\alpha}_h+{P}_{persist}{\beta}_h $$

Thus the fully extended scheme of fast inactivation including persistent currents and GS967 binding is given by

$$ A{\displaystyle \begin{array}{c}{\alpha}_h^{\hbox{'}}\\ {}\leftrightarrows \\ {}{\beta}_h^{\hbox{'}}\end{array}}I{\displaystyle \begin{array}{c}\mu \\ {}\leftrightarrows \\ {}\lambda \left[ GS967\right]\end{array}}{I}_{GS}. $$

Voltage step responses to typical voltage protocols (Fig. 5b-e) were calculated using custom simulation program MarkovEditor (http://users.ge.ibf.cnr.it/pusch/programs-mik.htm). These protocols included the standard IV pulses, and the protocol of recovery from fast inactivation. Holding voltage in these simulations was − 90 mV as in the experiments. In the program MarkovEditor, the kinetic equations are solved by calculation of eigenvectors and eigenvalues of the system of linear differential equations employing code from the Alglib C++ library (http://www.alglib.net/).

The model was then used to predict the effect of GS967 in a simplified single compartment neuronal model as in [10, 11] and using the program Neuron [31] to simulate the firing properties. In addition to the above described Na^+^ conductance, the neuronal model harbored a K^+^ conductance and a leak conductance with the same characteristics as described in [10, 11]. The simulated neuron had length and diameter of 25 μm, E_K_ = -85 mV. Simulations were performed for current injections between 10 and 350 pA (in 10 pA steps) and the membrane potential, the K^+^ current and the Na^+^ current were followed for 400 ms.

## Results

*Localization of FHM3 mutations in the Na* ^*+*^*channel structure*

**Fig. 1:**
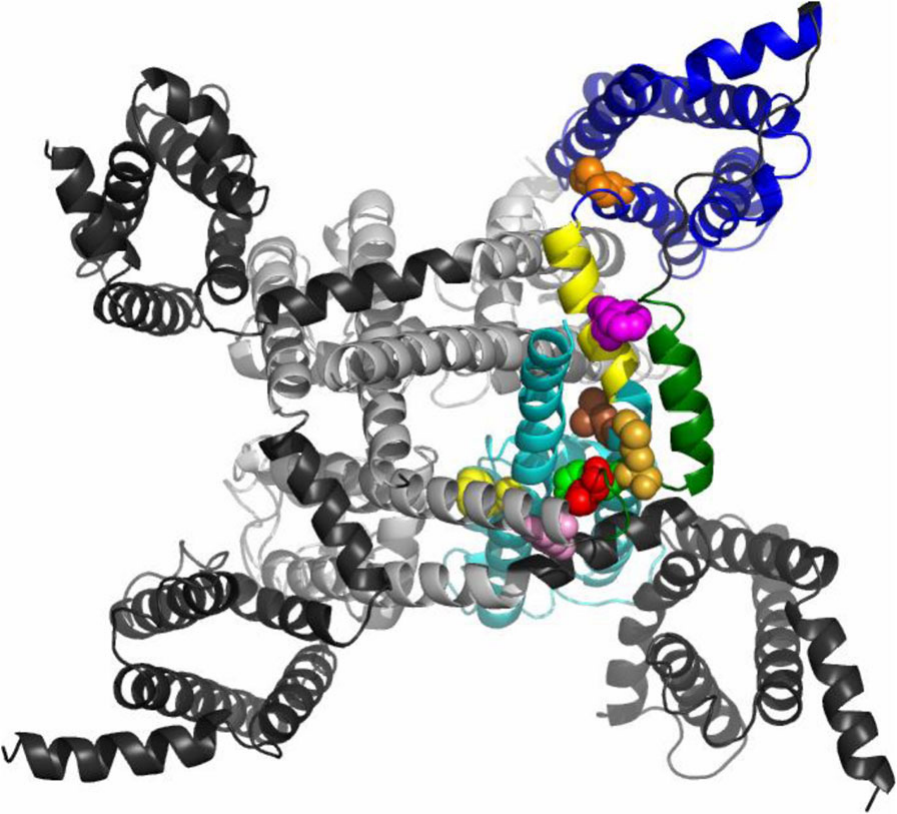
Mapping of mutants on the Na^+^ channel structure. The structure of the human skeletal muscle sodium channel Nav1.4 [32] is shown in cartoon structure. Domains I-III are shown in gray (voltage-sensor domains in dark gray, pore domains in light gray). The linker between domain III and IV is shown in green. For domain IV, the voltage sensor domain (S1-S4) is shown in dark blue, the pore domain in light blue and the linker S4-S5 in yellow. Residues corresponding to those studied here are shown in space fill: Q1489: light pink; I1498: red; F1499: green; M1500: light brown; L1649: orange; F1661: pink; F1774: yellow

Since FHM3 mutations affect critical biophysical functional properties of the voltage-gated sodium channel it is insightful to map their location onto the recently obtained Na^+^ channel protein structures. In Fig. 1 the residues corresponding to the mutations studied here are mapped onto the structure of the human skeletal muscle Na^+^ channel [32]. Interestingly, all mutations are localized in the fourth domain, which is implicated in the inactivation process [33]. All three residues of the most critical “IFM” motif [34] at the beginning of the loop connecting domains III and IV [35] are mutated in different patients (shown in space-fill representation in red, green and light-brown, respectively in Fig. 1). Thus, a priori, defects of the inactivation process are likely involved in FHM3. Q1489 (light pink in Fig. 1) is located slightly upstream at the end of S6 of domain III. F1661 (in pink in Fig. 1) is located in the middle of the S4-S5 linker of domain IV, which is shown in yellow cartoon representation. This linker helix is believed to couple voltage-sensor movements to pore opening. L1649 (orange) is in S4 of domain IV and F1774 (yellow) is in S6 of domain IV.

### Electrophysiological investigation of 5 uncharacterized FHM3 mutations

**Fig. 2:**
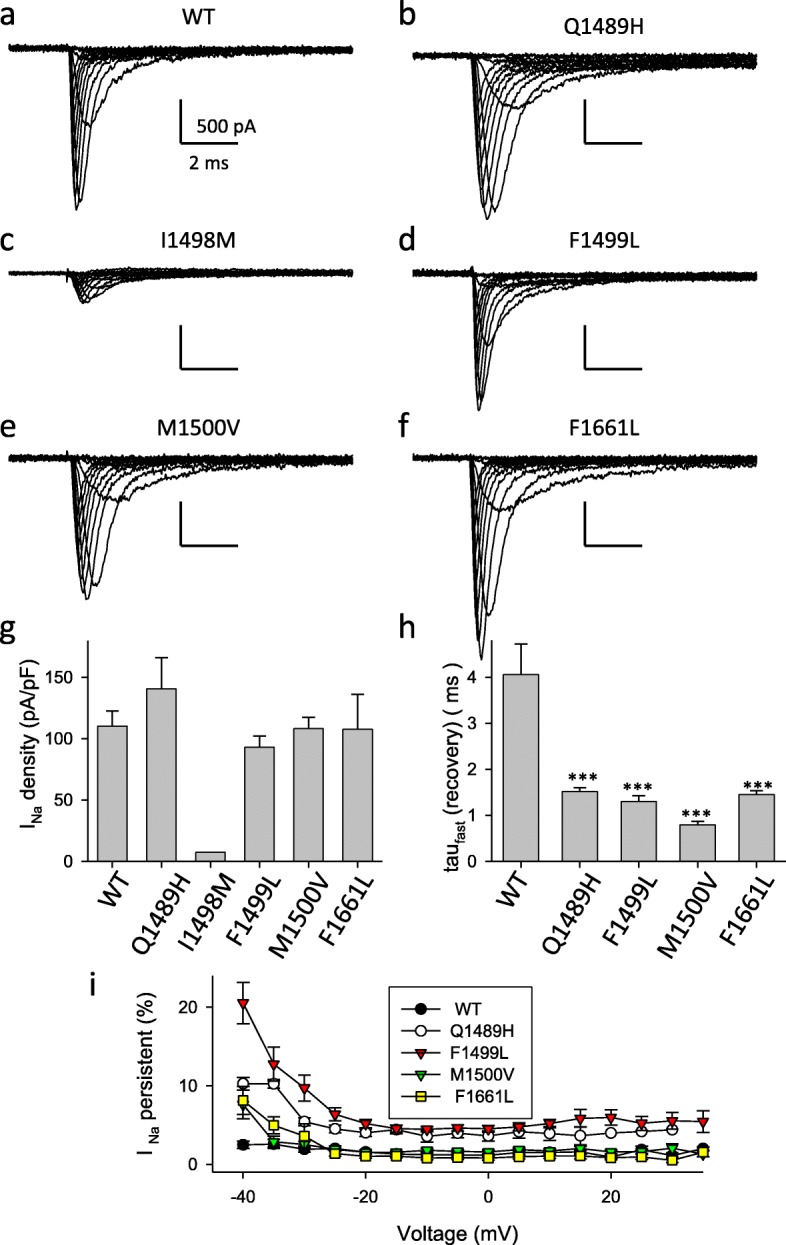
Electrophysiological analysis of 5 FHM3 mutations in HEK cells. **a**-**f** show typical voltage clamp current traces in response to the standard IV protocol. Scale bars: 2 ms and 500 pA, respectively. **g** shows the peak current density and **h** the time constant of recovery from inactivation at − 90 mV. **i** show the relative persistent current. Values for mutants F1499 L and Q1489H are significantly larger than those of WT (*p* < 0.001) at all voltages. Values for mutants F1661 L and M1500 V are statistically significantly different from those of WT only at − 40 mV (*p* < 0.05)

We introduced the following 5 FMH3 associated mutations into our optimized *SCN1A* expression plasmid: Q1489H and F1499 L were found in families in which FHM was associated with elicited repetitive daily blindness [36] (F1499 L is a recurrent mutation [37]); I1498M and F1661 L were found in patients with pure hemiplegic migraine [38]; M1500 V was detected by screening a cohort of patients with migraine with aura [39]. We first investigated these mutations in HEK cells using the patch clamp technique. While expression of Q1489H, F1499 L, M1500 V and F1661 L led to Na^+^ currents of practically the same size as WT, I1498M induced only small currents, rendering a detailed functional analysis practically impossible (Fig. 2 a-g; suppl. Table 1). Currents of I1498M could also not be rescued by incubation of cells at 30 °C (data not shown), a maneuver that had been successful for the L1649Q mutation [10]. For the other mutations the results of the quantitative analysis of gating properties are summarized in Fig. 2 h, i and Additional file 1: Table S1. Properties of activation and slow inactivation were rather similar to WT with small, but significant, shifts of the voltage of half-maximal activation for Q1489H and M1500 V, and slight changes in the times constant of the onset of slow inactivation for Q1489H, F1499 L, and F1661 L (Additional file 1: Table S1). In addition, none of the four mutations significantly altered the time constants of the onset of fast inactivation (data not shown). In contrast, other properties of the fast inactivation process were affected by all mutants in various proportions: they induced a shift of the voltage of half maximal inactivation to more positive values (Additional file 1: Table S1) and accelerated to various degrees the speed of recovery from inactivation (Fig. 2h, Additional file 1: Table S1). In addition, persistent currents were slightly enhanced in mutants M1500 V and F1661 L at − 40 mV, and markedly increased in mutants Q1489H and F1499 L (Fig. 2i). Thus, for all four mutants, the inactivation process is partially disrupted, a clear gain of function effect.

### Effect of GS967 on FHM3 mutations

Late Na^+^ current blockers have been proposed as possible treatment options for cardiac arrhythmia causing mutations of SCN5A that led to similar gain of function effects as seen here for *SCN1A* [17]. To evaluate if such a pharmacological strategy might be useful in FHM3, we tested the more recently discovered late Na^+^ current blocker GS967 on the above investigated four mutations (Q1489H, F1499 L, M1500 V, F1661 L) as well as on three previously characterized ones [14], most of which showed dramatic increases of persistent currents (L1649Q, L1670 W, F1774S). To this end, we expressed these mutants in *Xenopus* oocytes that allowed more stable recordings upon application of the drug compared to HEK cells. In addition, we exploited the fact that mutants L1649Q and L1670 W expressed rather large currents in oocytes, whereas these are difficult to express in HEK293 cells [8, 10, 13, 14]. This “rescue” is possibly caused by the lower incubation temperature of *Xenopus* oocytes (18 °C) compared to HEK cells (30-37 °C). Even though the absolute values of most gating parameters are slightly different in oocytes compared to HEK cells, the effects induced by the mutations are well reproduced in this system (Additional file 1: Table S3).

**Fig. 3:**
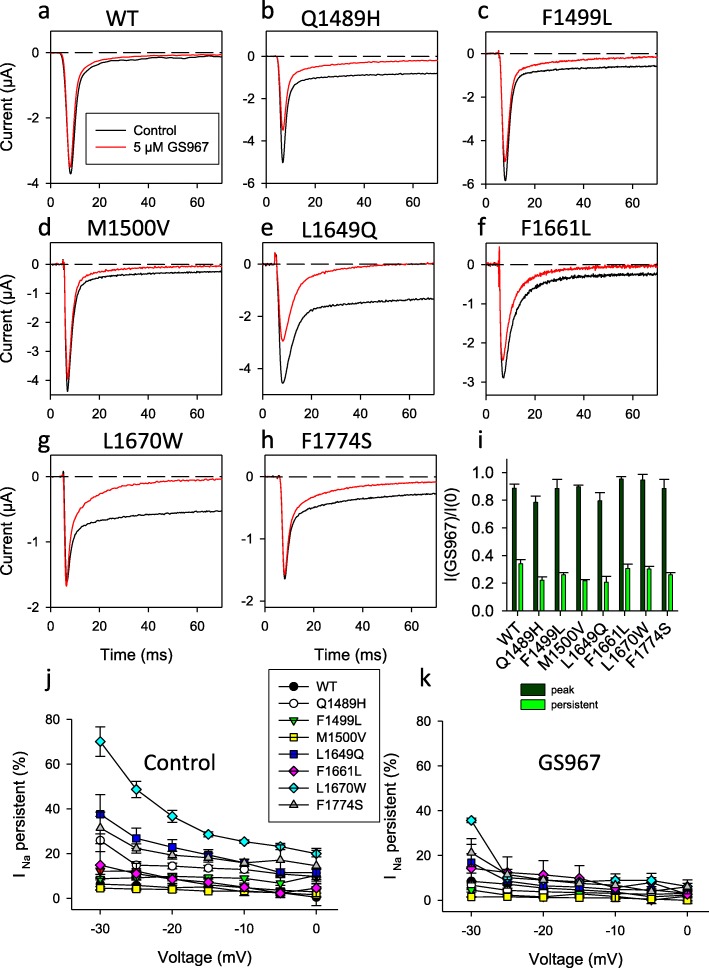
GS967 blocks persistent currents for WT and all mutants. **a**-**h** show typical voltage clamp current responses to a −30 mV (E), − 25 mV (A, B, C, D, F) or − 10 mV (G, H) pulse before (black traces) and after application of 5 μM GS967. The different voltages were chosen to maximize current amplitudes, which depended on the reversal potential. In particular, oocytes expressing mutants with large persistent currents had a negatively shifted reversal potential due to sodium accumulation. In panel **i** the average reduction induced by GS967 of peak currents and current at the end of the 70 ms test pulse is expressed as I (after GS967 application) / I (before application). **j** and **k** show relative persistent currents before (**j**) and after (**k**) application of GS967. GS967 significantly decreases persistent currents at all voltages (*p* < 0.05)

**Fig. 4:**
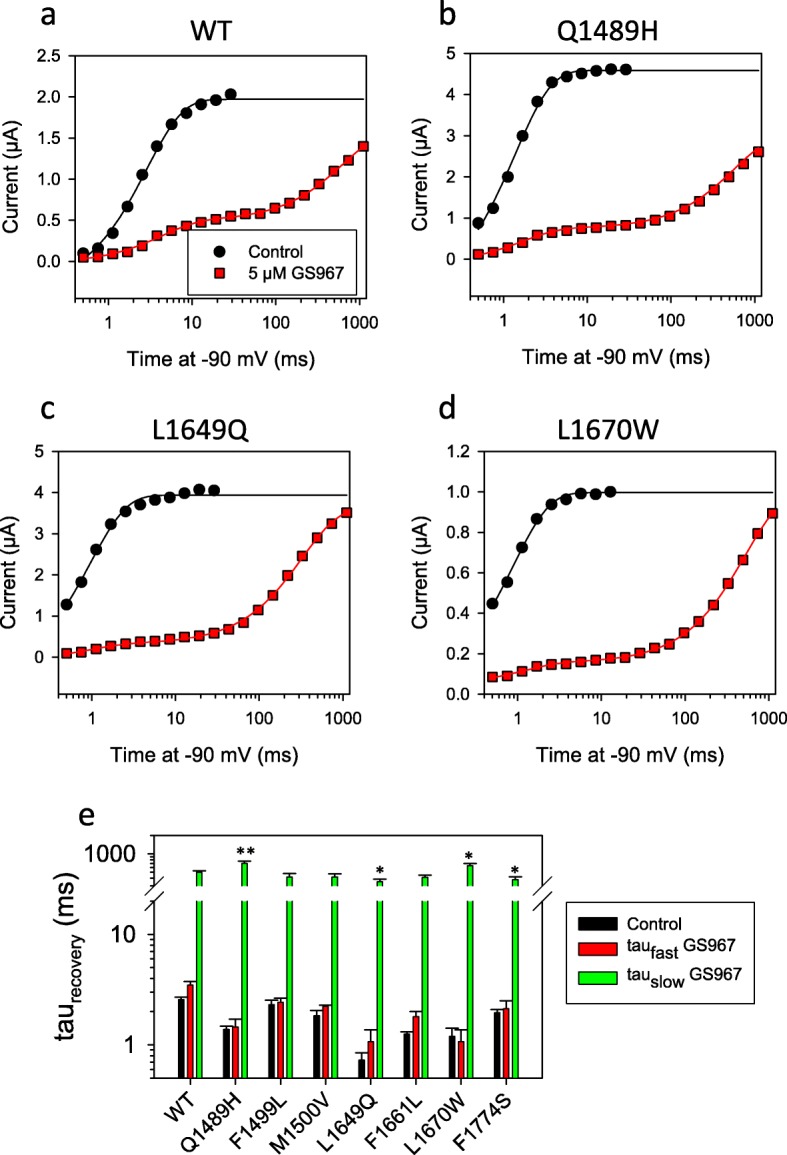
GS967 prolongs recovery from inactivation for WT and all mutants. **a**-**d** show typical results for the indicated constructs for measurements of the recovery from inactivation at − 90 mV before (black symbols) and after (red symbols) application of 5 μM GS967. Black lines are single exponential fits, red lines are fits of a double exponential function. **e** shows the average rime constants of recovery before and after application of GS967

The application of 5 μM GS967 slightly reduced peak currents but strongly decreased persistent currents in all mutants tested (Fig. 3 b-h, i-k), while showed only a small effect on WT (Fig. 3a) overall confirming that GS967 binds directly to and interacts with the Nav1.1 protein. Scrutinizing activation and inactivation properties revealed that GS967 had no effect on activation parameters and slightly shifted the voltage of half-maximal inactivation 2–5 mV to more negative values for all mutants, except for F1774S for which GS967 shifted the half maximal inactivation voltage by about − 16 mV (Additional file 1: Table S3). The most dramatic effect was seen for the process of recovery from inactivation, measured at − 90 mV. While WT and all mutants fully recovered within a few ms in the absence of GS967, the drug introduced an additional, and predominant, slow component with a time constant of 400–700 ms (Fig. 4). The concentration of GS967 used here was larger than that employed in studies that tested its antiarrhythmic effect in cardiac myocytes or isolated hearts [18]. However, this relatively high concentration was necessary in oocytes because smaller concentrations produced variable effects, in line with the general finding that hydrophobic drugs are less efficient in oocytes compared to mammalian cells, probably due to the unspecific binding to intracellular yolk (unpublished result). Control experiments on WT and mutant Q1489H in HEK293 cells reveled a clear block of persistent currents and stabilization of the recovery from inactivation also at 1 μM GS967 (data not shown), a concentration used in other patch clamp studies [40].

Overall the results on the effect of GS967 on FHM3 related mutations, suggest that this molecule specifically binds to and stabilizes the inactivated state of Nav1.1 channels.

### Simulation of the effect of GS967 on neuronal firing

**Fig. 5:**
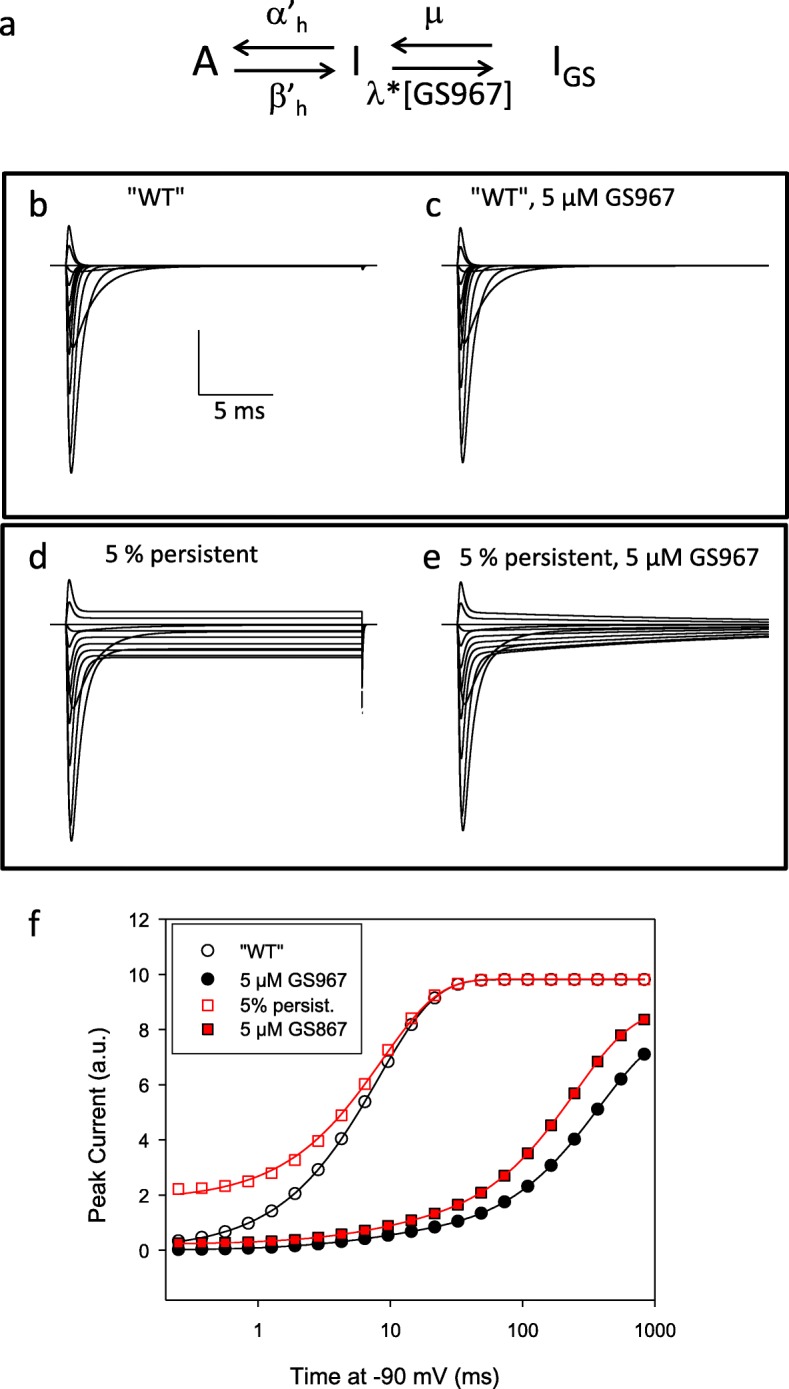
Incorporation of GS967 binding and persistent current in a Hodgkin-Huxley type model of Na^+^ channel gating. GS967 is assumed to bind exclusively to the inactivated state with association rate constant λ [GS967] and dissociation rate constant μ (**a**). The values (λ = 10 μM^− 1^ s^− 1^, μ = 1 s^− 1^) were chosen (but not optimized in any systematic manner) to reproduce in a qualitative manner the kinetic onset of GS967 binding during a prolonged depolarization, and to qualitatively reproduce the kinetics of recovery from inactivation. Predictions of the model in response to typical IV protocol are shown in B and C for channel with no persistent currents (“WT”) and in D and E for a channel with 5% persistent currents without (**b**, **d**) or with 5 μM GS967 (**c**, **e**). Panels E shows simulated results for the indicated conditions for the recovery from inactivation at − 90 mV (symbols) overlaid with single exponentials fits (without GS967) and double exponential fits (with GS967), respectively

In order to explore possible effects of GS967 on neural network firing, we extended previously used mathematical models of Na^+^ channel gating. In particular, in the framework of a classical Hodgkin-Huxley model [10, 11, 30] (see Methods for details) we hypothesized that GS967 can only bind to the inactivated state, extending the gating variable “*h*” as illustrated in Fig. 5a. Binding to inactivated channels occurs with second order association rate constant λ, while GS967 dissociates with rate constant μ. Figure 5b-e shows simulated voltage-clamp traces for “WT” (Fig. 5 b, c) or for “mutated” channels with persistent currents of 5% (Fig. 5 d, e), introduced in the model as described in Methods. Simulations were performed in the absence (Fig. 5 b, d) or in the presence of 5 μM GS957 (Fig. 5 c, e). For WT, currents in the presence of GS967 do not appear very different from those in the absence of the drug (Fig. 5c). For channels with 5% persistent currents, steady state currents are greatly diminished (Fig. 5e). For both channel types, the recovery from inactivation at − 90 mV after a 70 ms test-pulse to 0 mV is dramatically slowed by GS967 (Fig. 5f). These features reproduce well the experimental findings in a qualitative manner. Thus, even though the model itself is clearly simplified, it can be used to explore possible effects of the application of GS967 on the firing properties of a neuron with a Na^+^ conductance with increased persistent current.

**Fig. 6:**
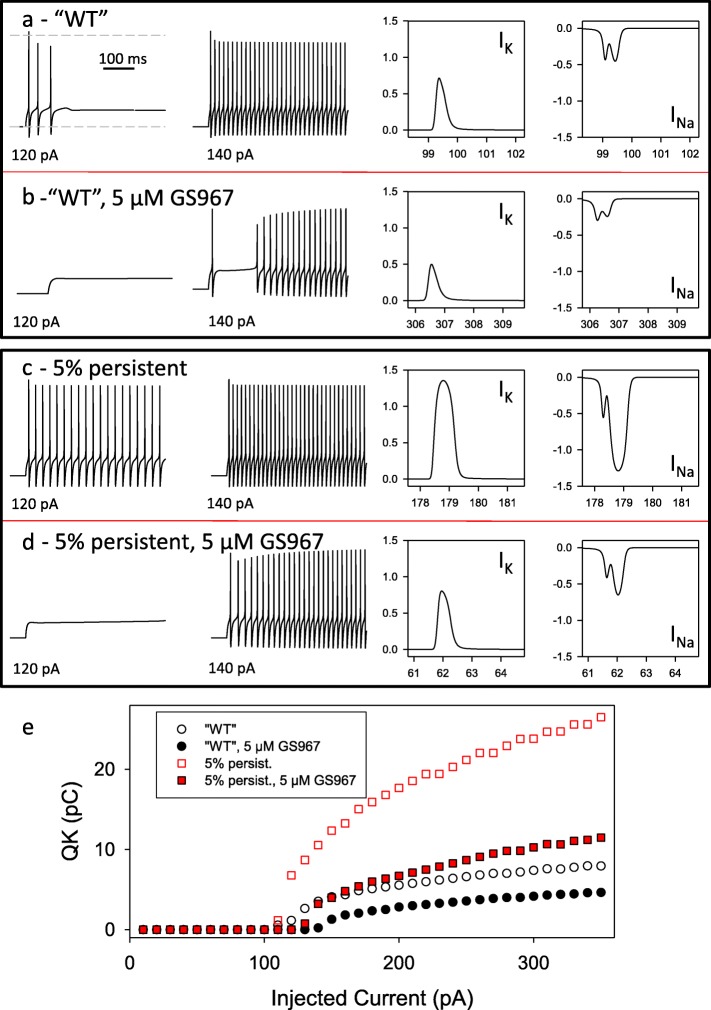
Simulation of neuronal firing. A single compartment neuron was simulated with the program Neuron as described in methods, with a “WT” like Na^+^ channel (without persistent currents) and with a Na^+^ channels with 5% persistent currents, each in the absence and presence of GS967. Each pane (**a**-**d**) shows on the left the membrane potential as a function of time for current injections of 120 and 140 pA, and on the right the K^+^ and Na^+^ currents of a single action potential. In panel **e** the integrated K^+^ efflux during the 400 ms stimulation period is shown as a function of the injected current for the indicated conditions

To this end we used a single compartment neuronal model as described in [10, 11] (see Methods for details) to assess the excitability as well as the overall K^+^ and Na^+^ current size during action potential firing. For a “WT” neuron, injection of 120 pA was necessary to elicit the first action potentials, and 140 pA triggered a continuous train of action potentials (Fig. 6a). Application of 5 μM GS957 slightly reduced excitability (Fig. 6b, left), but had only a modest effect on the size the associated ion currents (Fig. 6b, right panels). Note that the Na^+^ current is characterized by two peaks, corresponding to the depolarization and repolarization phases of the action potential, respectively. Increasing the percentage of the persistent current to 5% slightly increased excitability in that already injection of 120 pA led to continuous action potential bursting (Fig. 6c, left). Interestingly however, the ionic currents associated with each action potential were dramatically increased (Fig. 6c, right). For the Na^+^ current, the increase regarded mostly the second peak associated with repolarization. Again, application of 5 μM GS957 slightly decreased excitability (Fig. 6d, left). In addition GS967 led to a large reduction of the ionic currents associated with AP firing (Fig. 6d, right), acting mostly on the second peak of the Na^+^ current. The effect of GS967 on the overall charge transport associated with the action potential train is illustrated in Fig. 6e, which shows the integrated K^+^ current over a time period of 400 ms as a function of the injected current. An increase of the persistent current to 5% leads to a large increase in the K^+^ charge needed to repolarize the action potentials. Application of 5 μM GS967 dramatically reduces the K^+^ charge in mutated neurons to almost WT levels in the absence of GS967.

## Discussion

Our work concludes the investigation of all 12 so far identified FHM3 mutations in heterologous expression systems. We can now conclude that, among other small changes, for most mutations one or more gating parameters associated with the inactivation process are affected significantly in varying proportions, leading to reduced inactivation [2, 6–14]. In addition to defects in inactivation, some mutations were associated with a reduced expression level, including L1649Q and L1670 W [8, 10, 13, 14]. We now add the I1498M mutation to this list. In both HEK cells and oocytes only very small current levels could be detected for this mutant precluding a detailed functional analysis. From the few recordings we could obtain we can exclude that the mutant exhibits a large increase in persistent currents (see Fig. 2). However, more detailed analysis would be necessary to detect more subtle changes. Interestingly, the mutants L1649Q and L1670 W, which produce only small currents in HEK cells [8, 10, 13, 14], resulted in sizable current levels in oocytes, possibly caused by the lower incubation temperature. Unfortunately, this was not true for the I1498M mutant. In principle the small currents seen with mutation I1498M could be caused by a reduction of the single channel conductance, even though this is a priori not likely given that the affected residue is not close to the pore. Unfortunately, the currents were too small to allow a reliable estimate of the single channel conductance non-stationary noise analysis. In vivo studies of animal models will be needed to find out the degree of reduction of expression levels in the nervous system for the mutations with reduced expression in heterologous systems.

Defects in inactivation are predicted to result in hyperexcitability of interneurons as indeed suggested by previous simulations of neuronal firing [9–11, 13]. However, it is unclear if the increased hyperexcitability itself, i.e. increased firing rates, are responsible for the insurgence of migraine attacks. A further mechanism that could contribute to the triggering of migraine was suggested by the simulations of neuronal firing performed here. While the 3-conductance model used can reproduce trains of action potentials, it is clearly an extreme simplification of mammalian neuronal function. Therefore, the simulation results have no “literal” significance for a real neuron. Nevertheless, we found that introducing persistent currents leads to a dramatic increase of ion movements during the repolarization phase of the action potential. This is intuitively clear as a persistent Na^+^ conductance will lead to continuous Na^+^ inflow and consequently K^+^ outflow needed to repolarize the membrane potential. The increase in transmembrane ion movements is predicted to have two consequences. First, the extracellular K^+^ concentration will rise more than it would normally do during repetitive firing. Such an increase in extracellular K^+^ could be directly linked to the insurgence of spreading depression and migraine. More indirectly, increased K^+^ efflux and increased Na^+^ influx will increase the energy demands on the firing neuron itself and on surrounding astrocytes, needed to pump the excess K^+^ ions back into the intracellular space. If the energy demand exceeds the mitochondrial capacity of neurons / astrocytes, extracellular K^+^ will accumulate even more, resulting possibly in a catastrophic positive feedback loop. The effect might be attenuated by accumulation of intracellular Na^+^, which could lead to a reduction of persistent currents. However, as neurons are programmed to keep intracellular Na^+^ low, accumulation of Na^+^ actually adds to the energy burden. In this regard it is noteworthy that Hu et al. [41] found that the K^+^ and Na^+^ channels in fast spiking GABAergic interneurons, which are assumed to express predominantly *SCN1A*, are finely tuned to minimize the overlap of depolarizing Na^+^ current and repolarizing K^+^ current in order to minimize the energetic cost of action potential firing.

Late Na^+^ current blockers like ranolazine and GS967 have been proposed to be beneficial for treating patients that harbor inactivation deficient mutations in the cardiac *SCN5A* channels [17]. Ranolazine is certainly an interesting compound that has been shown to cross the blood-brain barrier [42]. Here, as a first attempt, since we are dealing with a neurological disease, we chose to test GS967, because this compound had been shown to reduce anomalous persistent currents in two mouse models affected by a severe encephalopathy (Nav1.6) and by Dravet syndrome (Nav1.1), respectively. In the latter case, GS967 significantly increased survival of affected animals [21, 23]. From these in vivo experiments, we could reasonably assume that GS967 permeates the blood-brain barrier as well and for this reason could be advantageous in FHM3 treatment.

We report for the first time that GS967 binds to Nav1.1 and potently and rather specifically inhibits the persistent current of FHM3 mutants with smaller effects on the peak currents for all mutants tested. Interestingly, residue F1774 (mutated here to S) corresponds to F1760 in *SCN5A* which has been implicated in local anesthetics binding [3]. However, Potet et al. found that F1760 is not essential for GS967 effects in *SCN5A* [40], in agreement with the potent reduction of persistent currents of F1774S seen here, suggesting that GS967 binds to a different site than local anesthetics.

Overall, the kinetic effects of GS967 are well explained by a preferential binding to the inactivated state of the channel, and indeed the recovery from inactivation is dramatically slowed by the drug. The effects found here are qualitatively very similar to those described for the cardiac Na^+^ channel [40]. These authors concluded that GS967, in addition to effects on fast inactivation, also alters slow inactivation [40]. However, possible effects on slow inactivation are confounded by the very slow recovery from fast inactivation. We could well model the effects of GS967 in a qualitatively manner for a WT like situation and for a situation with large persistent currents by the simplifying assumption that GS967 exclusively binds to the inactivated state, and, unless it dissociates, prevents recovery from inactivation. Using this model in a neuronal firing simulation suggested that application of GS967 could strongly reduce the above mentioned large ionic movements induced by large persistent currents.

## Conclusions

Our results support the hypothesis that FHM3 is caused by hyperactivity of the Nav1.1 channel due to a defect in the inactivation process and that GS967 may have beneficial effects inhibiting Nav1.1 persistent currents, which are greatly increased in FHM3 mutations. A preclinical testing of GS967 could be a valid approach to explore specific pharmacological treatment of FHM3. Genetic hemiplegic migraines are rare in themselves, but share many molecular mechanisms with the more common forms of migraine with or without aura. Thus, any compound that turns out to be a remedy for FHM3 could also have a therapeutic effect on common forms of migraine that affect a very large number of patients.

## Supplementary information

**Additional file 1.** Supplementary Methods and Tables

## Data Availability

The datasets analysed during the current study and the mutated SCNA1 clones are available from the corresponding author on reasonable request.

## References

[1] Pietrobon D, Moskowitz MA (2013). Pathophysiology of migraine. Annu Rev Physiol.

[2] Dichgans M, Freilinger T, Eckstein G, Babini E, Lorenz-Depiereux B, Biskup S (2005). Mutation in the neuronal voltage-gated sodium channel SCN1A in familial hemiplegic migraine. Lancet.

[3] Catterall WA (2014). Sodium channels, inherited epilepsy, and antiepileptic drugs. Annu Rev Pharmacol Toxicol.

[4] Escayg A, Goldin AL (2010). Sodium channel SCN1A and epilepsy: mutations and mechanisms. Epilepsia.

[5] Mantegazza M, Curia G, Biagini G, Ragsdale DS, Avoli M (2010). Voltage-gated sodium channels as therapeutic targets in epilepsy and other neurological disorders. Lancet Neurol.

[6] de Vries B, Freilinger T, Vanmolkot KRJ, Koenderink JB, Stam AH, Terwindt GM (2007). Systematic analysis of three FHM genes in 39 sporadic patients with hemiplegic migraine. Neurology.

[7] Vanmolkot KR, Babini E, de Vries B, Stam AH, Freilinger T, Terwindt GM, et al. The novel p.L1649Q mutation in the SCN1A epilepsy gene is associated with familial hemiplegic migraine: genetic and functional studies. Mutation in brief #957. Online. Hum Mutat 2007;28(5):522 10.1002/humu.9486 17397047

[8] Kahlig KM, Rhodes TH, Pusch M, Freilinger T, Pereira-Monteiro JM, Ferrari MD (2008). Divergent sodium channel defects in familial hemiplegic migraine. Proc Natl Acad Sci U S A.

[9] Cestèle S, Scalmani P, Rusconi R, Terragni B, Franceschetti S, Mantegazza M (2008). Self-limited hyperexcitability: functional effect of a familial hemiplegic migraine mutation of the Nav1.1 (SCN1A) Na^+^ channel. J Neurosci.

[10] Cestèle S, Schiavon E, Rusconi R, Franceschetti S, Mantegazza M (2013). Nonfunctional NaV1.1 familial hemiplegic migraine mutant transformed into gain of function by partial rescue of folding defects. Proc Natl Acad Sci U S A.

[11] Cestèle S, Labate A, Rusconi R, Tarantino P, Mumoli L, Franceschetti S (2013). Divergent effects of the T1174S SCN1A mutation associated with seizures and hemiplegic migraine. Epilepsia.

[12] Fan C, Wolking S, Lehmann-Horn F, Hedrich UB, Freilinger T, Lerche H et al (2016) Early-onset familial hemiplegic migraine due to a novel SCN1A mutation. Cephalalgia Epub 2016/01/15 10.1177/0333102415608360 PMC5105328 26763045

[13] Dhifallah S, Lancaster E, Merrill S, Leroudier N, Mantegazza M, Cestèle S (2018). Gain of Function for the SCN1A/hNav1.1-L1670W Mutation Responsible for Familial Hemiplegic Migraine. Front Mol Neurosci.

[14] Bertelli S, Barbieri R, Pusch M, Gavazzo P (2018). Gain of function of sporadic/familial hemiplegic migraine-causing SCN1A mutations: use of an optimized cDNA. Cephalalgia.

[15] Matthews E, Fialho D, Tan SV, Venance SL, Cannon SC, Sternberg D (2010). The non-dystrophic myotonias: molecular pathogenesis, diagnosis and treatment. Brain.

[16] Kroncke BM, Glazer AM, Smith DK, Blume JD, Roden DM (2018). SCN5A (NaV1.5) Variant Functional Perturbation and Clinical Presentation: Variants of a Certain Significance. Circ Genom Precis Med.

[17] Antzelevitch C, Burashnikov A, Sicouri S, Belardinelli L (2011). Electrophysiologic basis for the antiarrhythmic actions of ranolazine. Heart Rhythm.

[18] Belardinelli L, Liu G, Smith-Maxwell C, Wang WQ, El-Bizri N, Hirakawa R (2013). A novel, potent, and selective inhibitor of cardiac late sodium current suppresses experimental arrhythmias. J Pharmacol Exp Ther.

[19] Bossu A, Houtman MJC, Meijborg VMF, Varkevisser R, Beekman HDM, Dunnink A (2018). Selective late sodium current inhibitor GS-458967 suppresses Torsades de pointes by mostly affecting perpetuation but not initiation of the arrhythmia. Br J Pharmacol.

[20] Carneiro JS, Bento AS, Bacic D, Nearing BD, Rajamani S, Belardinelli L (2015). The selective cardiac late sodium current inhibitor GS-458967 suppresses Autonomically triggered atrial fibrillation in an intact porcine model. J Cardiovasc Electrophysiol.

[21] Anderson LL, Hawkins NA, Thompson CH, Kearney JA, George AL (2017). Unexpected efficacy of a novel Sodium Channel modulator in Dravet syndrome. Sci Rep.

[22] Anderson LL, Thompson CH, Hawkins NA, Nath RD, Petersohn AA, Rajamani S (2014). Antiepileptic activity of preferential inhibitors of persistent sodium current. Epilepsia.

[23] Baker EM, Thompson CH, Hawkins NA, Wagnon JL, Wengert ER, Patel MK (2018). The novel sodium channel modulator GS-458967 (GS967) is an effective treatment in a mouse model of SCN8A encephalopathy. Epilepsia.

[24] Koltun DO, Parkhill EQ, Elzein E, Kobayashi T, Notte GT, Kalla R (2016). Discovery of triazolopyridine GS-458967, a late sodium current inhibitor (late INai) of the cardiac NaV 1.5 channel with improved efficacy and potency relative to ranolazine. Bioorg Med Chem Lett.

[25] Günther W, Lüchow A, Cluzeaud F, Vandewalle A, Jentsch TJ (1998). ClC-5, the chloride channel mutated in Dent's disease, colocalizes with the proton pump in endocytotically active kidney cells. Proc Natl Acad Sci U S A.

[26] Moran O, Conti F, Tammaro P (2003). Sodium channel heterologous expression in mammalian cells and the role of the endogenous beta1-subunits. Neurosci Lett.

[27] Yan Zhen, Zhou Qiang, Wang Lin, Wu Jianping, Zhao Yanyu, Huang Gaoxingyu, Peng Wei, Shen Huaizong, Lei Jianlin, Yan Nieng (2017). Structure of the Na v 1.4-β1 Complex from Electric Eel. Cell.

[28] Hamill OP, Marty A, Neher E, Sakmann B, Sigworth FJ (1981). Improved patch-clamp techniques for high-resolution current recording from cells and cell-free membrane patches. Pflügers Arch.

[29] Vindas-Smith R, Fiore M, Vasquez M, Cuenca P, Del Valle G, Lagostena L (2016). Identification and functional characterization of CLCN1 mutationsg found in nondystrophic myotonia patients. Hum Mutat.

[30] Barela AJ, Waddy SP, Lickfett JG, Hunter J, Anido A, Helmers SL (2006). An epilepsy mutation in the sodium channel SCN1A that decreases channel excitability. J Neurosci.

[31] Hines ML, Carnevale NT (1997). The NEURON simulation environment. Neural Comput.

[32] Pan Xiaojing, Li Zhangqiang, Zhou Qiang, Shen Huaizong, Wu Kun, Huang Xiaoshuang, Chen Jiaofeng, Zhang Juanrong, Zhu Xuechen, Lei Jianlin, Xiong Wei, Gong Haipeng, Xiao Bailong, Yan Nieng (2018). Structure of the human voltage-gated sodium channel Nav1.4 in complex with β1. Science.

[33] Catterall WA (2000). From ionic currents to molecular mechanisms: the structure and function of voltage-gated sodium channels. Neuron.

[34] West JW, Patton DE, Scheuer T, Wang Y, Goldin AL, Catterall WA (1992). A cluster of hydrophobic amino acid residues required for fast Na(+)-channel inactivation. Proc Natl Acad Sci U S A.

[35] Stühmer W, Conti F, Suzuki H, Wang XD, Noda M, Yahagi N (1989). Structural parts involved in activation and inactivation of the sodium channel. Nature.

[36] Vahedi K, Depienne C, Le Fort D, Riant F, Chaine P, Trouillard O (2009). Elicited repetitive daily blindness: a new phenotype associated with hemiplegic migraine and SCN1A mutations. Neurology.

[37] Schubert V, Auffenberg E, Biskup S, Jurkat-Rott K, Freilinger T (2018). Two novel families with hemiplegic migraine caused by recurrent SCN1A mutation p.F1499L. Cephalalgia.

[38] Weller CM, Pelzer N, de Vries B, Lopez MA, De Fabregues O, Pascual J (2014). Two novel SCN1A mutations identified in families with familial hemiplegic migraine. Cephalalgia.

[39] Domitrz I, Kosiorek M, Zekanowski C, Kaminska A (2016). Genetic studies of polish migraine patients: screening for causative mutations in four migraine-associated genes. Hum Genomics.

[40] Potet F, Vanoye CG, George AL (2016). Use-dependent block of human cardiac sodium channels by GS967. Mol Pharmacol.

[41] Hu Hua, Roth Fabian C., Vandael David, Jonas Peter (2018). Complementary Tuning of Na+ and K+ Channel Gating Underlies Fast and Energy-Efficient Action Potentials in GABAergic Interneuron Axons. Neuron.

[42] Kahlig Kristopher M, Lepist Irene, Leung Kwan, Rajamani Sridharan, George Alfred L (2010). Ranolazine selectively blocks persistent current evoked by epilepsy-associated NaV1.1 mutations. British Journal of Pharmacology.

