# Supplementary material for: Late sodium current blocker GS967 inhibits persistent currents induced by familial hemiplegic migraine type 3 mutations of the SCN1A gene
Source: J Headache Pain. 2019 Nov 15;20(1):107. doi: 10.1186/s10194-019-1056-2 (PMC6858687; doi:10.1186/s10194-019-1056-2)
Supplement: Supplementary file 1 — Additional file 1. Supplementary Methods and Tables [file 10194_2019_1056_MOESM1_ESM.docx]

**Supplementary Methods.**

The following pulse protocols were applied to extract current size and gating parameters. For most protocols, linear leak and capacity was subtracted using standard P/4 leak subtraction. For protocols assessing parameters of slow inactivation, short “leak pulses” were applied and leak and capacitance were subtracted off-line.

The standard IV consisted of 20 ms pulses to voltages ranging from –50 mV to +40 mV. Steady-state activation was determined by fitting the peak current-voltage (I-V) relationship with the equation:

$$I\left( V \right)=\frac{g_{max}(V-V_{rev})}{1+{exp}^{-z_{act}(V{-V}_{1/2}^{act})F/RT}}$$

with *V_rev_* being the reversal potential, *g_max_* the maximum conductance, *V_1/2_^act^* the potential for half-maximal activation, *z_act_* the apparent gating valence, *R* the gas constant and *T* the temperature. The parameter *g_max_* is a measure of the functional expression level.

Persistent currents were assessed by a similar IV protocol with pulses of 70 ms duration. The relative persistent current was expressed as the ratio of the steady state current at the end of the test-pulse and the peak current.

For experiments in HEK cells, a quantitative analysis of the time course of inactivation was performed by fitting the decaying phase of the sodium current with a double exponential function of the form

$$I\left( t \right)=I_{\infty}+ I_{f}e^{-\frac{t}{\tau_{f}}}{+I}_{s}e^{-\frac{t}{\tau_{s}}}$$

with a “fast” time constant *τ_f_* and a “slow” time constant *τ_s_*.

Steady-state inactivation (channel availability) was measured after 100-ms conditioning pulses to various voltages followed at a test pulse to –10 mV, and currents were fitted with the equation:

$$I\left( V \right)=\frac{I_{max}}{1+{exp}^{z_{inact}(V{-V}_{1/2}^{inact})F/RT}}$$

where V represents the pre-pulse potential, $I_{max}$ is the maximal current, $V_{1/2}^{inact}$ the voltage of half-maximal inactivation, and z_inact_ the apparent gating valence of inactivation.

The time course of recovery from inactivation at -90 mV was measured by repolarizing the cell to -90 mV for a variable time after a 100 ms pulse to 0 mV, and assessing channel availability by a final test-pulse to -10 mV. Peak-currents at the final test pulse were analyzed by fitting an exponential function:

$$I\left( t \right)=I_{max}- I_{r}e^{-\frac{t}{\tau_{r}}}$$

where $\tau_{r}$ is time constant of recovery. Recovery from fast inactivation in HEK cells was also measured at -120 mV and required a bi-exponential fit with a prominent fast component. Also in the presence of GS967, recovery from inactivation at -90 mV had a bi-exponential time course and was thus fitted with a bi-exponential function:

$$I\left( t \right)=I_{max}- I_{rf}e^{-\frac{t}{\tau_{rf}}}{-I}_{rs}e^{-\frac{t}{\tau_{rs}}}$$

with a fast and a slow component of recovery.

In HEK cells we also assessed steady state slow inactivation, onset of slow inactivation at -10 mV and recovery from inactivation at -100 mV. For steady state slow inactivation, 20 s pulses to voltages ranging between -90 and -20 mV (in 10 mV steps) were followed by a 15 ms pulse to -100 mV (to remove fast inactivation) and a final test pulse to -10 mV. Peak currents during the final test pulses were fitted by the following equation

$$I\left( V \right)=\frac{I_{max}}{1+{exp}^{z_{s}(V{-V}_{1/2}^{s})F/RT}}$$

where V represents the pre-pulse potential, $I_{max}$ is the maximal current, $V_{1/2}^{s}$ the voltage of half-maximal slow inactivation, and z_s_ the apparent gating valence of slow inactivation.

Onset of slow inactivation was measured by applying progressively longer pre-pulses to -10 mV (durations between 0.1 s and 25.6 s), followed by a 15 ms pulse to -100 mV (to remove fast inactivation) and a final test pulse to -10 mV. Peak currents during the final test pulses were fitted by the following equation

$$I\left( t \right)=I_{0}e^{-\frac{t}{\tau_{os}}}$$

where $\tau_{os}$ is time constant of onset of slow inactivation.

Recovery from slow inactivation was measured by applying a 20 s inactivating pulse to -10 mV, followed by progressively longer pulses to -100 mV (durations between 0.1 s and 25.6 s), and a final test pulse to -10 mV. Peak currents during the final test pulses were fitted by the following equation

$$I\left( t \right)=I_{max}- I_{r}e^{-\frac{t}{\tau_{rs}}}$$

where $\tau_{rs}$ is time constant of recovery from slow inactivation.

Data analysis was performed using self-written software (Visual C++, Microsoft) and the SigmaPlot program (Systat Software, Inc., San Jose, CA).

**Supplementary Table 1.** Activation and inactivation parameters of mutants expressed in HEK cells. Errors indicate SEM; numbers in parenthesis indicate number of cells.

|  | Activation | | Inactivation | |
| --- | --- | --- | --- | --- |
|  | V_1/2_ (mV) | Z | V_1/2_ (mV) | z |
| WT | -25.3 ± 1.1 (27) | 4.9 ± 0.1 (27) | -58.3 ± 0.4 (19) | 4.5 ± 0.2 (19) |
| Q1489H | **-29.9 ± 1.6 (24)*** | 4.9 ± 0.2 (24) | **-51.5 ± 0.7 (17)***** | 4.7 ± 0.2 (17) |
| F1499L | -23.7 ± 0.8 (42) | 4.5 ± 0.1 (42) | **-48.7 ± 0.8 (56)***** | 4.2 ± 0.1 (56) |
| M1500V | **-28.9 ± 1.5 (28)*** | 5.1 ± 0.2 (28) | **-49.9 ± 0.7 (25)***** | 4.6 ± 0.1 (25) |
| F1661L | -24.7 ± 0.8 (9) | 4.4 ± 0.2 (9) | **-46.4 ± 1.7 (22)***** | 4.6 ± 0.1 (22) |

**Supplementary Table 2:** Time constants of recovery from inactivation and parameters of slow inactivation of mutants in expressed in HEK cells (time constants are in ms). Errors indicate SEM; numbers in parenthesis indicate number of cells.

|  | Recovery from inactivation | | Slow inactivation | | |
| --- | --- | --- | --- | --- | --- |
|  | τ_fast_ (-120 mV) | τ_slow_ (-120 mV) | V_1/2_ (mV) | τ_onset_ | τ_recovery_ |
| WT | 1.1 ± 0.1 (23) | 30.1 ± 2.5 (23) | -59.0 ± 1.89 (15) | 1138 ± 119 (8) | 7140 ± 1530 (11) |
| Q1489H | **0.55 ± 0.05 (19)***** | 32.4 ± 2.3 (19) | -59.5 ± 1.63 (15) | **598 ± 48 (14)***** | 5615 ± 1146 (8) |
| F1499L | **0.79 ± 0.07 (47)*** | 44.4 ± 3.5 (47) | -55.8 ± 0.99 (15) | **465 ± 32 (20)***** | 10150 ± 1125 (21) |
| M1500V | **0.70 ± 03 (26)**** | 28.0 ± 2.4 (26) | -56.0 ± 2.08 (14) | 1166 ± 102 (10) | 4134 ± 546 (6) |
| F1661L | **0.51 ± 0.02 (31)***** | **15.6 ± 0.9 (31)***** | -57.8 ± 2.08 (9) | **1623 ± 53 (9)*** | 7961 ± 1111 (22) |

**Supplementary Table 3.** Voltages of half-maximal activation and inactivation before (control) and after application of 5 µM GS967 of mutants expressed in oocyte. Significance is indicated for the comparison of mutant versus WT for the control conditions and control versus GS967 for the GS967 values. Values are in mV; errors indicate SEM; numbers in parenthesis indicate number of oocytes.

|  | Activation | | Inactivation | |
| --- | --- | --- | --- | --- |
|  | V_1/2_ control | V_1/2_ GS967 | V_1/2_ control | V_1/2_ GS967 |
| WT | -31.8 ± 0.8 (16) | -29.6 ± 0.8 (16) | -44.0 ± 0.7 (24) | -45.5 ± 0.8 (16) |
| Q1489H | -30.4 ± 1.0 (4) | -30.4 ± 2.6 (4) | **-**42.4 ± 1.2 (5) | -46.7 ± 1.6 (4) |
| F1499L | -33.3 ± 0.8 (12) | -34.2 ± 2.1 (12) | **-40.5 ± 1.3 (15)**** | -44.5 ± 1.8 (12) |
| M1500V | -32.1 ± 1.9 (4) | -33.3 ± 2.0 (4) | -42.1 ± 0.7 (4) | **-45.3 ± 0.7 (4)*** |
| L1649Q | -30.4 ± 2.8 (5) | -28.9 ± 2.5 (5) | **-32.4 ± 1.4 (6)***** | **-37.5 ± 1.5 (5)*** |
| F1661L | -34.2 ± 1.7 (13) | -32.6 ± 1.4 (11) | **-39.6 ± 0.8 (14)***** | **-42.3 ± 0.9 (12)*** |
| L1670W | **-18.0 ± 1.1 (13)***** | -16.2 ± 1.4 (9) | **-34.9 ± 1.6 (11)***** | -37.0 ± 0.8 (9) |
| F1774S | -26.9 ± 3.0 (7) | -29.0 ± 3.7 (7) | **-26.9 ± 3.0 (22)***** | **-42.1 ± 1.5 (9)***** |
